# Role of SLMAP genetic variants in susceptibility of diabetes and diabetic retinopathy in Qatari population

**DOI:** 10.1186/s12967-015-0411-6

**Published:** 2015-02-15

**Authors:** Rohit Upadhyay, Amal Robay, Khalid Fakhro, Charbel Abi Khalil, Mahmoud Zirie, Amin Jayyousi, Maha El-Shafei, Szilard Kiss, Donald J D′Amico, Jacqueline Salit, Michelle R Staudt, Sarah L O′Beirne, Xiaoliang Chen, Balwant Tuana, Ronald G Crystal, Hong Ding

**Affiliations:** Departments of Pharmacology, Weill Cornell Medical College-Qatar, Doha, Qatar; Departments of Genetic Medicine, Weill Cornell Medical College-Qatar, Doha, Qatar; Departments of Medicine, Hamad Medical Corporation, Doha, Qatar; Departments of Ophthalmology, Hamad Medical Corporation, Doha, Qatar; Departments of Ophthalmology, Weill Cornell Medical College, New York, NY USA; Departments of Genetic Medicine, Weill Cornell Medical College, New York, NY USA; Department of Cadre & Cardiology, The affiliated hospital of Hangzhou Normal University, Hangzhou, China; Faculty of Medicine, University of Ottawa, Ottawa, ON Canada

**Keywords:** SLMAP, Endothelial dysfunction, Genetic susceptibility, Qatari population, Diabetic retinopathy, T2DM

## Abstract

**Background:**

Overexpression of *SLMAP* gene has been associated with diabetes and endothelial dysfunction of macro- and micro-blood vessels. In this study our primary objective is to explore the role of *SLMAP* gene polymorphisms in the susceptibility of type 2 diabetes (T2DM) with or without diabetic retinopathy (DR) in the Qatari population.

**Methods:**

A total of 342 Qatari subjects (non-diabetic controls and T2DM patients with or without DR) were genotyped for *SLMAP* gene polymorphisms (rs17058639 C > T; rs1043045 C > T and rs1057719 A > G) using Taqman SNP genotyping assay.

**Results:**

*SLMAP* rs17058639 C > T polymorphism was associated with the presence of DR among Qataris with T2DM. One-way ANOVA and multiple logistic regression analysis showed *SLMAP* SNP rs17058639 C > T as an independent risk factor for DR development. *SLMAP* rs17058639 C > T polymorphism also had a predictive role for the severity of DR. Haplotype C_rs17058639_T_rs1043045_A_rs1057719_ was associated with the increased risk for DR among Qataris with T2DM.

**Conclusions:**

The data suggests the potential role of *SLMAP* SNPs as a risk factor for the susceptibility of DR among T2DM patients in the Qatari population.

**Electronic supplementary material:**

The online version of this article (doi:10.1186/s12967-015-0411-6) contains supplementary material, which is available to authorized users.

## Introduction

Currently, 387 million people worldwide have diabetes, which is approximately 8.3% of the total adult population and in 2014 diabetes contributed to 4.9 million deaths [1]. Three of the world’s top 10 countries with the highest prevalence (%) of diabetes are Saudi Arabia, Kuwait, and Qatar [2]. In Qatar, the prevalence of diabetes is increasing (16.28% vs 8.5% global) with the total diabetic population expected to increase by 130% by 2030 [1-3].

Consistently high blood glucose levels associated with type 2 diabetes (T2DM) can lead to serious disabling and life-threatening complications including retinopathy. The mechanism of hyperglycemia induced retinal microvascular damage is not clear, however, multiple interconnected pathways including polyol pathway, activation of protein kinase C (PKC), increased vascular endothelial growth factor (VEGF) and insulin-like growth factor-1 (IGF-1), accelerated formation of advanced glycation endproducts (AGEs), oxidative stress, activation of the renin-angiotensin-aldosterone system (RAAS), and subclinical inflammation and capillary occlusion have been proposed [4]. Diabetes is responsible for approximately 12% of all causes of blindness [5], and diabetic retinopathy (DR) prevalence among diabetes patients has been estimated 34.6% [6-9]. About 1/3 of patients with T2DM develop signs of DR, and a 1/3 of these are afflicted with vision-threatening retinopathy [6], although there is a disparity in the risk of T2DM/ DR between different ethnic groups [10]. The Qatari population has one of the highest prevalence rates for diabetes, but has not been explored genetically.

The state of glycemic control, blood pressure and duration of diabetes plays a significant role on the impact of development and progression of DR; however, other factors including genetic variations also contribute to the development of T2DM and associated DR as evident from previous literature [10-12]. Recently Rodriguez-Flores *et al.* [13]*,* assessed 100 Qataris by exome sequencing and reported many unique variations as well as a higher frequency of variants in comparison with *1000 Genomes* populations, suggesting that the Qatari population may have unique genetic associations for disease risk. Qatari individuals can be subdivided into three clearly definable subpopulations: Q1-Bedouin, Q2-Persian- South Asian, and Q3-African ancestry [14,15]. Each of these subpopulations has been shown to have a high degree of consanguinity [13,16].

With this background, the focus of this study was to assess the genetic variations of *SLMAP* (sarcolemma associated protein; OMIM ID: 602701) gene in the Qatari population with and without T2DM. *SLMAP* is a protein-coding gene associated with frozen shoulder, Becker muscular dystrophy, Brugada syndrome and diabetes (www.genecards.org) [17-19]. *SLMAP* is localized to chromosome 3p21.2-p14.3 in the human genome, a region that is also enriched in genes linked to T2DM [20-22]. *SLMAP* has 26 exons that encode several isoforms with 21 splice variants via alternative splicing [22,23]. Although the functional involvement of SLMAP in diabetic pathophysiology is still under investigation, SLMAP expression levels have been linked to vascular dysfunction in diabetes [18], and studies using diabetic *db/db* and diabetic *Tally Ho* mice suggest that deregulation of SLMAP expression may play an important role in T2DM [18,24].

**Figure 1:**
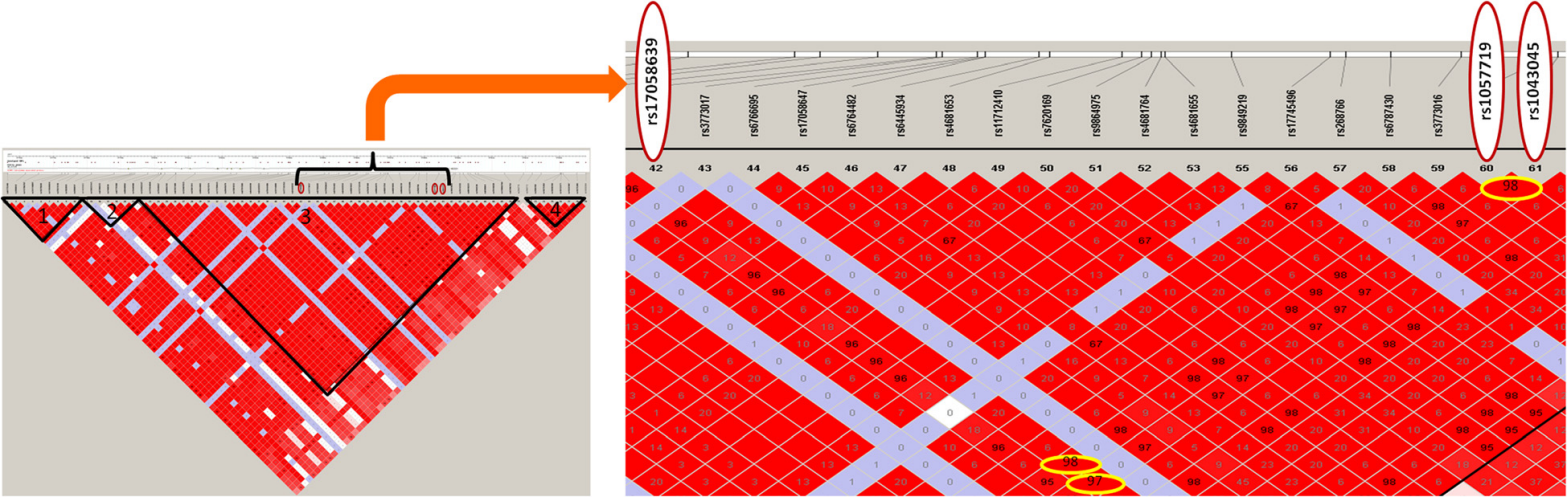
**Haplotype organisation of** ***SLMAP*** **gene and selected SNPs.** Haplotype organisation of *SLMAP* gene in the “CEU + TSI” population; The haplotype block pattern was constructed using the Haploview v4.1 using data from the HapMap project; Haploblocks were defined by four gamete rule; Measures of linkage disequilibrium (r^2^) among selected (three) *SLMAP* SNPs is shown by encircling the values in haploblock 3 (rs17058639: rs1057719 = 0.98; rs17058639: rs1043045 = 0.97; rs1057719: rs1043045 = 0.98).

The role of *SLMAP* gene polymorphisms in susceptibility of diabetes (with or without retinopathy) has not been explored. In this article, we aimed to assess the contribution of *SLMAP* gene polymorphisms to the susceptibility of T2DM with or without DR and their association with clinical phenotypes in the Qatari population. Three *SLMAP* SNPs [*SLMAP* rs17058639 C > T; *SLMAP* rs1043045 C > T and *SLMAP* rs1057719 A > G]; representing the major haplogroup of the gene (Figure 1) and predicted to have functional roles: either by abolishing protein domain through splicing (rs17058639 C > T) or due to binding of micro RNA in an allele specific manner (rs1043045 C > T/ rs1057719 A > G); were evaluated in this study.

## Materials and methods

### Study subjects

A total of 342 Qatari subjects were recruited for this study from the clinics of Hamad Medical Corporation (HMC) and HMC satellite clinics in Doha, Qatar. Subjects were individually interviewed with approved questionnaire for ethnicity, family history of disease and other demographic/clinical details. Subjects with incomplete clinical information/diagnosis were excluded. The parameters assessed included age, gender, fasting glucose levels, body-mass index (BMI), creatinine levels, glycated hemoglobin (HbA1C) levels, high density lipid (HDL)/ low density lipid (LDL) concentration, triglyceride level, blood urea nitrogen level (BUN), presence/absence of DR, DR subtypes, family history of disease, usage of oral hypoglycemic or insulin, and occurrence of gestational diabetes. According to the clinical diagnosis subjects were divided into three groups: Non-diabetic controls (NDC), T2DM patients without retinopathy (DNR) and diabetic patients with retinopathy (DR). The diagnosis of DR patients was done by standard clinical procedures and retinal phenotypes and verified by two independent ophthalmologists at, clinics of Hamad Medical Corporation (HMC), Doha, Qatar and Weill Cornell Medical College in New York City, USA. In this study the total number of subjects in NDC, DNR and DR groups was 104, 160 and 78 respectively.

Among the NDC group, ~50% of subjects had BMI >30; however their fasting glucose, triglycerides, and other metabolic markers were normal. This type of subjects usually referred as the “metabolically healthy obese” (MHO) and are assumed to be at no higher risk for T2DM or cardiovascular disease (CVD) than their metabolically healthy lean counterparts [25]. A fraction of subjects (N = 36) selected for present study was previously assessed for exome sequencing by Rodriguez-Flores et al. [13]. All of the subjects were third generation Qataris and whose ancestors were Qatari citizens by birth. Recent immigrants or residents of Qatar who traced their recent ancestry to other geographic regions were excluded. Each individual was genotyped for a panel of 48 SNPs through TaqMan (Life Technologies, Carlsbad, CA) to determine Q1, Q2, or Q3 ancestry in a Qatari individual [14]. Approximately 95% individuals in the present study had Q1 ancestry. Human subjects were recruited and written informed consents were obtained at HMC, Doha, Qatar. The study protocol was approved by the joint Hamad-Weill Cornell-Qatar Medical Research Center & Research Committee and the Institutional Review Board of Weill Cornell Medical College in Qatar (WCMC-Q).

### Genotyping

DNA was isolated from blood samples using QIAamp DNA blood kit following the manufacturer’s instructions (Qiagen, Valencia, CA, USA). The qualities of isolated genomic DNAs were checked using Nanodrop Analyzer (ND-1000) spectrophotometer (Nano Drop Technologies, Inc., Wilmington, DE, USA). The ratio of absorbance of DNA at 260 and 280 nm was 1.7–1.9. Isolated DNA was stored at −20°C. All SNP genotyping were performed using the Taqman SNP genotyping assay (Applied Biosystems Inc., Foster City, CA, USA). Reactions were carried out according to the manufacturer’s protocol. The probe fluorescence signal detection was performed using the ABI step-one fast Real-Time PCR System.

### Statistical analysis

Sample size calculations were done through Quanto ver. 1.2.4 software [26]. Diabetic prevalence in Qatar was taken as 16% in the total population; threshold level for significance was set at 0.05 and MAF was assumed ~30% (Additional file 1: Table S1; minimum global MAF was >30%). Upon putting these parameters to check a relative risk of 2.0 with Quanto ver. 1.2.4 software, we concluded that a study of 76 cases and 76 controls had 80% power in a log additive model to detect a SNP with the disease-causing variant.

Data were analyzed using SPSS 15.0 (Chicago, IL, USA). The χ^2^ goodness-of-fit test was used to determine differences in genotype and allele frequencies and deviation from Hardy–Weinberg equilibrium. Descriptive statistics of patients (T2DM with or without DR) and controls are presented as mean and standard deviation for continuous measures, or as frequency and percentage for categorical measures. The significance level for all statistical tests was set at P < 0.05. P-values were corrected (Pcorr) for multiple comparisons (Bonferroni correction) in case of further subgrouping or stratification. An independent t-test was applied to compare means of continuous variables; however, chi-square P-value was estimated for categorical variables. Logistic regression was used to calculate the odds ratio for various predictors, adjusting for the confounding effects of age and gender. The homozygous genotype for the low risk predicted allele in the control group (or DNR group for DR group) was used as the reference in calculating odds ratios (OR) for T2DM groups “with or without” DR and 95% confidence intervals (95% CI). P_trend_ analysis was performed to detect a disease severity dependent risk trend with genotypes. Analysis for the recessive model was also performed, where homozygous and heterozygous low risk (predicted) alleles were clubbed and taken as reference.

To detect association of genetic polymorphism with continuous clinical variables, one-way ANOVA analysis was performed. Mean values of continuous variables were compared among *SLMAP* genotypes and P value was estimated. To predict the cumulative risk variables in overall study multiple regression analysis was performed. “t statistics” in multiple regression analysis was used to identify significant predictors of the disease in the study. Significance was determined when the “t values” was well below −2 or above +2 and P value was <0.05.

Haplotypes were constructed through SNP-Analyzer 2.0 [27]. D′ and r^2^ values were calculated as measure indices of linkage disequilibrium (LD). LD plot was constructed and r^2^ values (for Hapmap populations) were obtained through “Haploview” software. Furthermore, disease risk due to haplotypes was calculated by logistic regression analysis through SPSS 15.0.

## Results

### SNP selection and function prediction

The *SLMAP* gene spans approximately 173.72 kb (57755450 bp to 57929168 bp) has a total of 3108 SNPs; none have previously been explored in T2DM or associated complications. We selected functional polymorphisms of *SLMAP*, through two SNP function predictor and prioritization software “SNP-info” and “Fast SNP” [28,29], having a minor allele frequency (MAF) >20% to get appropriate power of study. In the present study, after screening all SNPs of SLMAP gene, we selected three SNPs: rs17058639 C > T, rs1043045 C > T and rs1057719 A > G.

*SLMAP* rs17058639 C > T is a synonymous coding region polymorphism (FS score 0.910) predicted to be involved in splicing of *SLMAP* gene; rs1043045 C > T and rs1057719 A > G are located at 3`UTR region and predicted to have allele specific binding with hsa-miR-936 and hsa-miR-197 respectively (both have FS score 0.101) (Additional file 1: Table S1). Selected all three SNPs were located in the 3^rd^ haplogroup of *SLMAP* gene (Figure 1). This haplogroup is the major haplogroup of *SLMAP* gene which covers 110 kb region of total 173.72 kb gene.

### Clinical and demographic characteristics of study subjects

T2DM subjects with or without retinopathy had higher BMI and increased level of glucose, glycated hemoglobin A1c, HDL, triglycerides compared to NDC. There were no differences in these parameters among T2DM patients with or without retinopathy (Table 1). DR patients had significantly increased HbA1c, blood urea nitrogen, creatinine, HDL/LDL ratio, as well as increased insulin usage compared to DNR. Also, T2DM patients with or without retinopathy had a higher prevalence of a family history of diabetes in first-degree relative compared to controls (NDC = 11.7%, DNR = 28.4%%, DR = 24.4%, P value: NDC vs DNR = 0.00011; NDC vs DR = 0.00023).

**Table 1:** **Demographic and clinical characteristics of study subjects (N = 342)**

**Characteristics**	**Non diabetic controls (NDC) (N = 104)**	**T2DM patients without DR (DNR) (N = 160)**	**P value**	**T2DM patients with DR (N = 78)**	**P value**	**P value**
			**NDC vs DNR**		**NDC vs DR**	**DNR vs DR**
Mean Age (Yrs ± SD)	46.81 ± 10.32	55.64 ± 10.23	**4.702 ×10** ^**−10**^	57.86 ± 8.67	**1.403 ×10** ^**−11**^	0.101
Gender: Males	44 (42.3%)	58 (36.3%)	0.3252	36 (46.2%)	0.6073	>0.05
Gender: Females	60 (57.7%)	102 (63.8%)		42 (53.8%)		
Mean Glucose levels (mmole/lit) ± SD	5.36 ± 0.97	10.55 ± 6.68	**2.5×10** ^**−8**^	9.92 ± 4.23	**1.2 × 10** ^**−12**^	0.491
BMI (mean)	30.41 ± 6.70	34.08 ± 6.99	**5.3 × 10** ^**−5**^	33.92 ± 6.53	**0.001**	0.867
Creatinine level (micromole/lit) ± SD	72.44 ± 36.81	69.30 ± 28.55	0.529	81.5 ± 45.27	0.236	**0.025**
Glycated hemoglobin (HbA1c) mean% ± SD (mmol/mol)	5.63 ± 0.42 (38)	8.24 ± 1.72 (67)	**5.2 × 10** ^**−9**^	8.82 ± 1.83 (73)	**4.39 × 10** ^**−10**^	**0.032**
Total Cholesterol (mmol/lit) mean ± SD	5.07 ± 0.87	4.88 ± 0.99	0.253	4.62 ± 1.08	**0.022**	0.105
HDL (mmol/lit) mean ± SD	1.40 ± 0.34	1.22 ± 0.34	**0.003**	1.21 ± 0.34	**0.008**	0.930
LDL (mmol/lit) mean ± SD	3.11 ± 0.77	2.95 ± 0.94	0.299	2.68 ± 1.04	**0.021**	0.085
HDL/LDL ratio (mean ± SD)	0.48 ± 0.18	0.45 ± 0.19	0.457	0.55 ± 0.33	0.186	**0.015**
Triglycerides (mmol/lit) mean ± SD	1.24 ± 0.56	1.77 ± 1.23	**0.006**	1.62 ± 0.75	**0.005**	0.394
Blood Urea Nitrogen (mmol/lit) mean ± SD	5.14 ± 2.42	5.24 ± 2.54	0.793	6.97 ± 4.49	**0.007**	**0.001**
DR subtypes: No DR: BDR/NPDR: PDR:DME	104:0:0:0	160:0:0:0	NA	0:19(24.4%):10(12.8%):49(62.8%)	NA	NA
Presence of 1° Family history of diabetes	4/109 (3.67%)	45/160 (28.4%)	**1.86 × 10** ^**−6**^	19/78 (24.4%)	**0.0002**	0.641
Usage of Hypoglycemics	0	136 (85.0%)	NA	63 (80.8%)	NA	0.457
Usage of Insulin	0	66 (41.3%)	NA	56 (71.8%)	NA	**9.36 × 10** ^**−6**^
Fasting plasma glucose ≥7 mM		60 (37.5%)	NA	23 (29.5%)	NA	0.248
Gestational diabetes in females (n;%)	7 (11.7%)/60	48 (47.06%)/102	**0.00011**	22 (52.4%)/42	**0.00023**	0.714
Diabetes occurrence after pregnancy	0/65	28 (27.5%)/102	NA	14 (33.3%)/42	NA	0.546

NDC = Non diabetic controls; T2DM = Type 2 Diabetes mellitus; DNR = Diabetic non-retinopathy; DR = Diabetic retinopathy; NPDR = Non-proliferative diabetic retinopathy; PDR = Proliferative diabetic retinopathy; BDR = Background diabetic retinopathy; DME = Diabetic macular edema; NA = not applicable; significant values shown in bold.

### Frequency distribution and association of *SLMAP* gene polymorphisms with T2DM with or without retinopathy

Assessment of the MAF of selected *SLMAP* gene polymorphisms in different ethnic groups/populations (based on Hapmap data; http://hapmap.ncbi.nlm.nih.gov) demonstrated that the frequencies of the selected SNPs were heterogeneous among different populations, but there was no data from the Qatari population (Additional file 1: Table S1). The present study comprised 109 NDCs, approximately 50% of whom were metabolically healthy obese (MHO). When we stratified NDC according to their BMI (<30 vs >30) we did not observe any significant association with *SLMAP* gene polymorphisms (data not shown). Chi square test showed genotypic distribution of *SLMAP* rs17058639 C > T, rs1043045 C > T and rs1057719 A > G polymorphisms were in agreement with Hardy-Weinberg equilibrium in controls (P_HWE_ > 0.05). There was no significant difference in distribution of *SLMAP* rs17058639 C > T genotypes among T2DM patients and NDCs. However, frequency of the CC genotype was significantly higher in DR compared to DNR (53.8% vs 37.5%, Table 2). When Binary logistic regression was applied, *SLMAP* rs17058639 C > T polymorphism was not associated with risk of T2DM but *SLMAP* rs17058639 CC genotype significantly conferred enhanced risk of retinopathy in T2DM patients in homozygote and recessive models (CC vs TT: OR = 3.23, 95% CI = 1.22-8.52. P = 0.018; CC vs CT + TT: OR = 1.76, 95% CI = 1.15-2.68, P = 0.009). In allele contrast model, *SLMAP* 1705863 C allele was also significantly associated with higher risk of retinopathy in T2DM patients (OR = 1.76, 95% CI = 1.15-2.68, P = 0.009). For the other two *SLMAP* gene polymorphisms (rs1043045 C > T and rs1057719 A > G) there was no association with T2DM alone or with DR (Table 2).

**Table 2:** **Frequency distribution and association of** ***SLMAP*** **gene polymorphisms with T2DM/DR**

***SLMAP*** **gene polymorphisms**	**Non diabetic controls (NDC) (N = 104)**	**T2DM patients without DR (DNR) (N = 160)**	OR* (95% CI) P	**T2DM patients with DR (N = 78)**	OR* (95% CI)P
			**Controls vs T2DM**		**DNR vs DR**
*SLMAP* (rs17058639)C > T	N (%)	N (%)		N (%)	
TT	11 (10.6%)	29 (18.1%)	Reference	6 (7.7%)	Reference
CT	49 (47.1%)	71 (44.4%)	0.60 (0.26-1.39) 0.231	30 (38.5%)	2.08 (0.77-5.60) 0.146
CC	44 (42.3%)	60 (37.5%)	0.52 (0.22-1.22) 0.136	42 (53.8%)	**3.23 (1.22-8.52) 0.018**
TT + CT	60 (57.7%)	100 (62.5%)	Reference	36 (46.2%)	Reference
CC	44 (42.3%)	60 (37.5%)	0.77 (0.45-1.34) 0.357	42 (53.8%)	**1.82 (1.04-3.18) 0.035**
T	71 (34.1%)	129 (40.3%)	Reference	42 (26.9%)	Reference
C	137 (65.9%)	191 (59.7%)	0.75 (0.51-1.12) 0.160	114 (73.1%)	**1.76 (1.15-2.68) 0.009**
*SLMAP* (rs1043045)C > T					
CC	11 (10.6%)	31 (19.4%)	Reference	9 (11.5%)	Reference
CT	51 (49.0%)	70 (43.8%)	0.56 (0.24-1.28)0.167	31 (39.7%)	1.57 (0.66-3.73) 0.306
TT	42 (40.4%)	59 (36.9%)	0.51 (0.22-1.19) 0.120	38 (48.7%)	2.09 (0.89-4.92) 0.090
C	73 (35.1%)	132 (41.3%)	Reference	49 (31.4%)	Reference
T	135 (64.9%)	188 (58.8%)	0.76 (0.51-1.12) 0.162	107 (68.6%)	1.46 (0.97-2.20)0.068
*SLMAP* (rs1057719)A > G					
GG	11 (10.6%)	30 (18.8%)	Reference	8 (10.3%)	Reference
AG	50 (48.1%)	71 (44.4%)	0.59 (0.22-1.20) 0.215	32 (41.0%)	1.73 (0.71-4.23) 0.226
AA	43 (41.3%)	59 (36.9%)	0.51 (0.22-1.20) 0.125	38 (48.7%)	2.28 (0.94-5.53) 0.070
G	72 (34.6%)	131 (40.9%)	Reference	48 (30.8%)	Reference
A	136 (65.4%)	189 (59.1%)	0.75 (0.50-1.11) 0.150	108 (69.2%)	1.49 (0.99-2.24) 0.058

NDC = Non diabetic controls; T2DM = Type 2 Diabetes mellitus; DNR = Diabetic non-retinopathy; DR = Diabetic retinopathy; *Age and gender adjusted odds ratio and p-value; significant values shown in bold.

Stratification of subjects based on their gender indicated that *SLMAP* rs17058639 C > T polymorphism might increase the risk of DR specifically in male subjects in recessive and allele contrast models (CC vs CT + TT: OR = CI = 2.45, 95% CI = 1.03-5.84, P = 0.042; C allele vs T allele: OR = 2.16, 95% CI = 1.09-4.27, P = 0.028); however, upon applying Bonferroni correction for multiple comparisons P value did not reach the threshold of significance (Additional file 1: Table S2). There was no gender specific association of *SLMAP* rs1043045 and rs1057719 polymorphisms in DNR or DR groups (Additional file 1: Table S2).

### Association of *SLMAP* rs17058639 polymorphisms with clinical phenotypes and progression of DR

Since only *SLMAP* rs17058639 polymorphism was associated with the risk of DR, we also analyzed its interaction with various clinical parameters through one-way ANOVA analysis. The *SLMAP* rs17058639 polymorphism did not affect any of the clinical characteristics suggesting it as an independent risk predictor (Additional file 1: Table S3). The association of *SLMAP* rs17058639 polymorphism was also evaluated with prognosis of DR by comparing its frequency with different severity levels of DR. We performed “trend analysis” and found a significant trend of association between *SLMAP* rs17058639 CC genotype and diabetic macular edema (DME) suggesting a role as a prognostic marker for DR in T2DM patients (Additional file 1: Table S4). There was a non-significant trend for proliferative diabetic retinopathy (PDR).

### Haplotype construction and their association with risk of T2DM with or without retinopathy

Figure 1 shows haplotype organisation of the *SLMAP* gene in the “CEU + TSI” population which was constructed using the Haploview v4.1 software using data from the HapMap project. Haploblocks were defined by four gamete rule and the r^2^ value among selected *SLMAP* SNPs was >0.80 (rs17058639:rs1057719 = 0.98; rs17058639: rs1043045 = 0.97; rs1057719: rs1043045 = 0.98). Haplotype analysis in present study (Qatari population) also showed that all three selected *SLMAP* polymorphisms were in complete LD with each other in controls (│D'│ = 1, P value = 0.0001; r^2^= >0.80) (Additional file 1: Table S5; Figure 1). Four haplotypes were constructed. Haplotype combination C _rs17058639_T _rs1043045_A _rs1057719_ (CTA) and C_rs17058639_C _rs1043045_G _rs1057719_ (CCG) were associated with increased risk of retinopathy in T2DM patients compared to haplotype T _rs17058639_C _rs1043045_G _rs1057719_ (TCG) (OR = 1.79, 95% CI = 1.17-2.74, P value = 0.007; OR = 11.01, 95% CI = 2.20-55.11, P value = 0.005; respectively). Also, when risk haplotypes were combined together, haplotype combination CTA + CCG + CCA conferred a two-fold increased risk of retinopathy in T2DM in comparison with TCG haplotype (OR = 1.90, 95% CI = 1.25-2.90, P value = 0.003) (Table 3).

**Table 3:** ***SLMAP*** **haplotype frequency and association with the risk of DR development in T2DM patients**

**Haplotypes**	**Non diabetic controls (NDC = 208)**	**T2DM patients without DR (DNR =320)**	OR* (95%CI) P	**T2DM patients with DR (N = 156)**	OR8* (95% CI) P
	**N (%)**	**N (%)**	**Controls vs T2DM**	**N (%)**	**DNR vs DR**
T _rs17058639_C _rs1043045_G _rs1057719_	71 (34.14)	129 (40.31)	Reference	41 (26.28)	Reference
C _rs17058639_T _rs1043045_A _rs1057719_	135 (64.90)	188 (58.75)	0.77 (0.53-1.10) 0.153	107 (68.59)	**1.79 (1.17-2.74) 0.007**
C _rs17058639_C _rs1043045_G _rs1057719_	1 (0.48)	2 (0.63)	1.10 (0.10-12.35) 0.938	7 (4.49)	**11.01 (2.20-55.11) 0.004**
C _rs17058639_C _rs1043045_A _rs1057719_	1 (0.48)	0	ND	1 (0.64)	ND
**Grouped analysis**					
T _rs17058639_C _rs1043045_G _rs1057719_	71 (34.1)	129 (40.4)	Reference	41 (26.3)	Reference
CTA + CCG + CCA	137 (65.9)	190 (59.6)	0.76 (0.53-1.10)	115 (73.7)	**1.90 (1.25-2.90) 0.003**

NDC = Non diabetic controls; T2DM = Type 2 Diabetes mellitus; DNR = Diabetic non-retinopathy; DR = Diabetic retinopathy; ND = not determined; *Age and gender adjusted odds ratio and p-value; significant values shown in bold.

### Multiple regression analysis for detecting cumulative DR susceptibility predictors in the study

Since multiple clinical parameters and *SLMAP* rs17058639 C > T polymorphisms were identified as independent risk factors for DR susceptibility among T2DM patients, we checked for a holistic effect of selected *SLMAP* gene polymorphisms and clinical parameters on risk of DR. Multiple regression analysis showed *SLMAP* rs17058639 C > T gene polymorphism together with HbA1c and LDL were significant modulators of DR in T2DM patients (Table 4).

**Table 4:** **Prediction of variable in study for the risk of developing DR in T2DM patients by multiple regression analysis**

**Variables**	**T2DM patients without DR (DNR)**	**T2DM patients with DR**	**t-statistics value (t)**	**P value**
	**N (%)**	**N (%)**		
Age (mean ± SD)	55.64 ± 10.23	57.86 ± 8.67	0.29	0.776
Gender (Males vs Females)	58 (36.3%)/102 (63.8%)	36 (46.2%)/42 (53.8%)	1.13	0.262
BMI (mean ± SD)	34.08 ± 6.99	33.92 ± 6.53	−0.463	0.644
Creatinine level (micromole/lit) ± SD	69.30 ± 28.55	81.5 ± 45.27	1.82	0.070
Glycated hemoglobin (HbA1c) mean% ± SD	8.24 ± 1.72	8.82 ± 1.83	**1.99**	**0.048**
LDL(mmol/lit)mean ± SD	2.95 ± 0.94	2.68 ± 1.04	**−2.22**	**0.028**
Triglycerides (mmol/lit) mean ± SD	1.77 ± 1.23	1.62 ± 0.75	−1.07	0.286
Blood Urea Nitrogen (mmol/lit) mean ± SD	5.24 ± 2.54	6.97 ± 4.49	−0.944	0.347
Mean Glucose levels (mmole/lit) ± SD	10.55 ± 6.68	9.92 ± 4.23	−0.165	0.87
*SLMAP* rs17058639 C > T genotypes	TT = 29 (18.1%)	TT = 6 (7.7%)	**−2.15**	**0.033**
	CT = 71 (44.4%)	CT = 30 (38.5%)		
	CC = 60 (37.5%)	CC = 42 (53.8%)		
*SLMAP* rs1043045 C > T	CC = 31 (19.4%)	CC = 9 (11.5%)	0.95	0.294
	CT = 70 (43.8%)	CT = 31 (39.7%)		
	TT = 59 (36.9%)	TT = 38 (48.7%)		
*SLMAP* rs1057719 A > G	GG = 30 (18.8%)	GG = 8 (10.3%)	0.10	0.319
	AG = 71 (44.4%)	AG = 32 (41.0%)		
	AA = 59 (36.9%)	AA = 38 (48.7%)		

t= > +2 or < −2 depicts significance in data; significant values shown in bold.

## Discussion

Overexpression of SLMAP correlates with endothelial dysfunction in the microvasculature of diabetic db/db mice [18], and several other studies have suggested the role of SLMAP in diabetes and other macro/micro vascular diseases [30-32]. In the present study we have explored the genetic association of *SLMAP* gene polymorphisms [*SLMAP* rs17058639 C > T, rs1043045 C > T and rs1057719 A > G] in the Qatari population, which has one of the highest incidence of diabetes worldwide. We have observed for the first time that *SLMAP* rs17058639 C > T polymorphism is associated with risk of DR among T2DM patients in the Qatari population.

*SLMAP* genetic polymorphisms have heterogeneity in their frequency distribution among different ethnic groups. Upon comparing Hapmap data with present study; MAF of *SLMAP* gene polymorphisms in Qatari population was comparable with Mexican (MEX, M) population (Additional file 1: Table S1) which is also a high risk population for DR. Haffner et al. have showed significant two fold higher risk of proliferative DR in Mexican Americans compared to non-Hispanic whites after adjusting all other significantly different predictors [33]. These links suggest a possible similar *SLMAP* genetic association with DR in Mexican population; however future multi-ethnic studies will be needed to come at any conclusion.

Approximately fifty percent of NDCs in the present study were obese, but we did not find any difference between genotype frequencies of *SLMAP* gene polymorphisms and BMI. We did not observe any significant association of *SLMAP* gene polymorphisms between T2DM without or with DR and NDC. However, *SLMAP* rs17058639 C > T polymorphism was significantly associated with DR in comparison to T2DM without DR. These results indicate that *SLMAP* rs17058639 C > T polymorphism may contribute to the risk of DR but not to the susceptibility for T2DM.

Genetic variations may act as protective or risk factors; but no definitive genetic associations with DR have been consistently reported in the literature [34]. Candidate gene analysis and systematic genome wide association studies (GWAS) have identified a number of potential susceptibility genes for DR e.g.: *NOS2A, Bgl II, PAI-1, APOE, MTHFR, SUV39H2, IGF-1, PEDF, ICAM-1* and *TCF7L2* [10,35,36]. The three candidate genes that have been extensively studied and further analyzed by association studies for their possible influence on DR are vascular endothelial growth factor (VEGF), aldose reductase (ALR), and the receptor for advanced glycation end products (RAGE) [10,37].

We found that *SLMAP* rs17058639 C > T gene polymorphism might be associated with increased risk of DR in male subjects but in the present study the association did not reach statistical significance. The literature indicates that the risk for T2DM was female gender biased 50 years ago but now due to the increase in sedentary life style in men the risk of T2DM has shifted towards male gender [38]. In Iran, the risk of DR has been reported to be almost the same for both genders [39]. Since many known clinical risk factors for DR development were significantly elevated in the present study we therefore have evaluate if any of these parameters have an association with *SLMAP* rs17058639 C > T. One-way ANOVA analysis revealed that *SLMAP* rs17058639 C > T genotypes do not influence any of the clinical parameters significantly, suggesting that *SLMAP* (rs17058639) C > T polymorphism may be an independent predictor of DR. Multiple logistic regression analysis showed that HbA1c%, LDL levels and *SLMAP* (rs17058639) C > T gene polymorphisms were the main predictors for DR risk among T2DM patients. This genetic polymorphism was also found to be associated with the trend of DR progression; but due to smaller sample sizes in the sub grouped cells P values were either marginally significant or statistically non-significant.

The mechanistic reason for the significant association of *SLMAP* rs17058639 C > T polymorphism with DR is unknown but the *SLMAP* rs17058639 C > T polymorphism (Exon 16 synonymous Asp447Asp) is presented at a site for exonic splicing enhancer (ESE), a DNA sequence motif consisting of 6 bases within an exon that directs or enhances accurate splicing of hetero-nuclear RNA (hnRNA) or pre-mRNA into messenger RNA (mRNA). ESE is clinically significant because synonymous point mutations previously thought to be silent mutations located in an ESE can lead to exon skipping and production of a non-functional protein. The C allele is ancestral and has an active site for ESE but its substitution by the T allele due to SNP (rs17058639) may lead to production of non-functional or downregulated SLMAP protein which may further reduce the risk of T2DM or associated complications since the SLMAP expression has been shown to be elevated in microvascular diseases [18].

We did not observe any significant association with the other two 3′ UTR region *SLMAP* gene polymorphisms selected in this study; however, the difference in the allelic frequency among NDC, DNR and DR was obvious. *SLMAP* rs1043045 C > T and *SLMAP* rs1057719 A > G polymorphisms have potential predicted functional role in SLMAP expression. SNP function prediction by SNPinfo has showed that *SLMAP* rs1043045 C allele and *SLMAP* rs1057719 G alleles have binding sites for hsa-miR-936 and hsa-miR-197 respectively, but the substitution with polymorphic allele disrupts the binding of miRs. Allele specific binding of miRs reduce the expression of SLMAP therefore *SLMAP* rs1043045 C and *SLMAP* rs1057719 G alleles may function as protective alleles in diabetic subjects. To detect any synergistic effect of these two 3′ UTR region SNPs with codonic SNP (rs17058639) for DR risk, haplotypes were constructed and linkage disequilibrium was estimated. All three SNPs were in complete linkage in controls and haplotypes C_rs17058639_T_rs1043045_A_rs1057719_ and C_rs17058639_C_rs1043045_G_rs1057719_ were found significantly associated with DR risk in T2DM patients. This finding conferred that *SLMAP* 3`UTR SNPs selected in this study independently have no significant effect, but in combination with *SLMAP* rs17058639 C > T polymorphism these may increase the susceptibility of DR among T2DM patients.

The limitation of the present study is the small sample size, especially in the subgroups; therefore the findings should be interpreted cautiously and need to be considered as exploratory. Nonetheless, the novel findings presented here provide the basis for future similar and replicative studies in larger cohorts.

## Conclusions

In conclusion, this exploratory study in the unique high-risk population for T2DM (Qatari population) indicates novel association of *SLMAP* gene polymorphisms with DR risk among T2DM patients. *SLMAP* SNP rs17058639 C > T emerged as an independent risk factor for DR risk; however, *SLMAP* rs1043045 C > T and *SLMAP* rs1057719 A > G polymorphisms synergistically contributed to the increased risk of DR among T2DM patients. Therefore, *SLMAP* gene polymorphisms may be novel genetic predictors for the risk of DR in the Qatari diabetic population.

## Additional file

Additional file 1: Table S1. Details of *SLMAP* gene polymorphisms selected in the study. **Table S2.** Gender specific risk of DR with *SLMAP* polymorphisms. **Table S3.** Association of *SLMAP* rs17058639 C>T genotypes with clinical phenotypes (one-way ANOVA analysis). **Table S4.** Association of *SLMAP* rs17058639 C>T genotypes with progression of diabetic retinopathy. **Table S5.** Pair-wise linkage disequilibrium (Test of linkage disequilibrium for all pairs of loci).

## Abbreviations

SLMAP: Sarcolemma associated protein
T2DM: Type 2 Diabetes mellitus
DR: Diabetic retinopathy
NDC: Non-diabetic controls
DNR: Diabetic non-retinopathy
LD: Linkage disequilibrium
MAF: Minor allele frequency
NPDR: Non-proliferative diabetic retinopathy
PDR: Proliferative diabetic retinopathy
BDR: Background diabetic retinopathy
DME: Diabetic macular edema
