# Supplementary material for: Role of SLMAP genetic variants in susceptibility of diabetes and diabetic retinopathy in Qatari population
Source: J Transl Med. 2015 Feb 15;13:61. doi: 10.1186/s12967-015-0411-6 (PMC4335364; doi:10.1186/s12967-015-0411-6)
Supplement: Additional file 1: Table S1. — Details of SLMAP gene polymorphisms selected in the study. Table S2. Gender specific risk of DR with SLMAP polymorphisms. Table S3. Association of SLMAP rs17058639 C>T genotypes with clinical phenotypes (one-way ANOVA analysis). Table S4. Association of SLMAP rs17058639 C>T genotypes with progression of diabetic retinopathy. Table S5. Pair-wise linkage disequilibrium (Test of linkage disequilibrium for all pairs of loci). [file 12967_2015_411_MOESM1_ESM.docx]

Supplementary Table S1 Details of *SLMAP* gene polymorphisms selected in study

| ***SLMAP* gene polymorphisms** | **Gene location** | **MAF in different populations** | **Function Prediction by “SNP info” and F-SNP** |
| --- | --- | --- | --- |
| *SLMAP*  rs17058639 C>T | Codon | Global= 31.2%  ASW (A)=18.4%  CEU (C)=27.2%  CHB (H)= 48.2%  CHD (D)=49.5%  GIH (G)=35.1%  JPT (J)= 56.2%  LWK (L)= 5.5%  MEX (M)= 34.8%  MKK (K)= 11.2%  TSI (T)= 26.0%  YRI (Y)= 18.2%  Qatari population (present study)= 34.1% | ESE binding site  Splicing regulation |
| *SLMAP*  rs1043045 C>T | 3`UTR | Global= 36.4%  ASW (A)=35.7%  CEU (C)=26.5%  CHB (H)= 48.9%  CHD (D)=48.6%  GIH (G)=35.0%  JPT (J)= 52.7%  LWK (L)= 28.6%  MEX (M)= 33.6%  MKK (K)= 28.2%  TSI (T)= 26.0%  YRI (Y)= 43.8%  Qatari population (present study)= 35.1% | hsa-miR-936 binding site; transcriptional regulation |
| *SLMAP*  rs1057719 A>G | 3`UTR | Global= 35.9%  ASW (A)=34.2%  CEU (C)=27.0%  CHB (H)= 47.8%  CHD (D)=48.6%  GIH (G)=35.1%  JPT (J)= 52.7%  LWK (L)= 23.9%  MEX (M)= 34.5%  MKK (K)= 24.4%  TSI (T)= 26.5%  YRI (Y)= 42.9%  Qatari population (present study)= 34.6% | hsa-miR-197 binding site; transcriptional regulation |

Underlined nucleotides represent minor alleles; MAF= Minor allele frequency; Global= 1000 Genomes data; ASW (A) = African ancestry in southwest USA; CEU (C)=Utah residents with northern and western ancestry from the CEPH collection; CHB (H)= Han Chinese in metropolitan Denver, Colorado; GIH (G)= Gujarati Indians in Houston, Texas; JPT (J)= Japanese in Tokyo, Japan; LWK (L)= Luhya in Webuye, Kenya; MEX (M)= Mexican ancestry in Los Angeles, California

Supplementary Table S2: Gender specific risk of DR with *SLMAP* polymorphisms

|  | **Non diabetic controls (NDC)**  **N (%)** | **T2DM patients without DR (DNR)**  **N (%)** | **OR^‡^ (95%CI) P**  **Ctrl vs DNR** | **T2DM patients with DR**  **N (%)** | **OR^‡^ (95% CI)P**  **DNR vs DR** |
| --- | --- | --- | --- | --- | --- |
| ***SLMAP***  **rs17058639 C>T** |  |  |  |  |  |
| **Males** | N=44 | N=58 |  | N=36 |  |
| TT | 2 (4.5%) | 10 (17.2%) | Reference | 3 (8.3%) | Reference |
| CT | 24 (54.5%) | 22 (37.9%) | 0.23 (0.04-1.21) 0.082 | 9 (25.0%) | 1.38 (0.30-6.27) 0.677 |
| CC | 18 (40.9%) | 26 (44.8%) | 0.29 (0.05-1.55) 0.147 | 24 (66.7%) | 3.09 (0.76-12.60) 0.116 |
| CT+TT | 26 (59.1%) | 32 (55.2%) | Reference | 12 (33.3%) | Reference |
| CC | 18 (40.9%) | 26 (44.8%) | 0.98 (0.42-2.28) 0.962 | 24 (66.7%) | **2.45 (1.03-5.84) 0.042^†^** |
| **Alleles** | N=88 | N=116 |  | N=72 |  |
| T | 28 (31.8%) | 42 (36.2%) | Reference | 15 (20.8%) | Reference |
| C | 60 (68.2%) | 74 (63.8%) | 0.76 (0.41-1.42)0.395 | 57 (79.2%) | **2.16 (1.09-4.27) 0.028^††^** |
| **Females** | N=60 | N=102 |  | N=42 |  |
| TT | 9 (15.0%) | 19 (18.6%) | Reference | 3 (7.1%) | Reference |
| CT | 25 (41.7%) | 49 (48.0%) | 0.85 (0.31-2.34) 0.755 | 21 (50.0%) | 2.46 (0.65-9.34) 0.187 |
| CC | 26 (43.3%) | 34 (33.3%) | 0.63 (0.23-1.77) 0.381 | 18 (42.9%) | 3.05 (0.79-11.89) 0.107 |
| CT+TT | 34 (56.7%) | 68 (66.7%) | Reference | 24 (57.1%) | Reference |
| CC | 26 (43.3%) | 34 (33.3%) | 0.71 (0.34-1.47)0.355 | 18 (42.9%) | 1.47 (0.70-3.11) 0.309 |
| **Alleles** | N=120 | N=204 |  | N=84 |  |
| T | 43 (35.8%) | 87 (42.6%) | Reference | 27 (32.1%) | Reference |
| C | 77 (64.2%) | 117 (57.4%) | 0.77 (0.46-1.29)0.321 | 57 (67.9) | 1.52 (0.88-2.61) 0.133 |
| ***SLMAP***  **rs1043045 C>T** |  |  |  |  |  |
| **Males** | N=44 | N=58 |  | N=36 |  |
| CC | 2 (4.5%) | 10 (17.2%) | Reference | 5 (13.9%) | Reference |
| CT | 25 (56.8%) | 22 (37.9%) | 0.22 (0.04-1.16) 0.073 | 10 (27.8%) | 0.92 (0.25-3.41) 0.895 |
| TT | 17 (38.6%) | 26 (44.8%) | 0.30 (0.06-1.64) 0.166 | 21 (58.3%) | 1.62 (0.48-5.46) 0.440 |
| **Alleles** | N=88 | N=116 |  | N=72 |  |
| C | 29 (33.0%) | 42 (36.2%) | Reference | 20 (27.8%) | Reference |
| T | 59 (67.0%) | 74 (63.8%) | 0.80 (0.43-1.49) 0.489 | 52 (72.2%) | 1.47 (0.78-2.80)0.238 |
| **Females** | N=60 | N=102 |  | N=42 |  |
| CC | 9 (15.0%) | 21 (20.6%) | Reference | 4 (9.5%) | Reference |
| CT | 26 (43.3%) | 48 (47.1%) | 0.80 (0.29-2.16)0.655 | 21 (50.0%) | 2.14 (0.65-7.09) 0.214 |
| TT | 25 (41.7%) | 33 (32.4%) | 0.59 (0.21-1.64) 0.313 | 17 (40.5%) | 2.50 (0.73-8.57) 0.145 |
| **Alleles** | N=120 | N=204 |  | N=84 |  |
| C | 44 (36.7%) | 90 (44.1%) | Reference | 29 (34.5%) | Reference |
| T | 76 (63.3%) | 114 (55.9%) | 0.75 (0.45-1.25) 0.268 | 55 (65.5%) | 1.45 (0.85-2.47)0.177 |
| ***SLMAP***  **(rs1057719)A>G** |  |  |  |  |  |
| **Males** | N=44 | N=58 |  | N=36 |  |
| GG | 2 (4.5%) | 10 (17.2%) | Reference | 4 (11.1%) | Reference |
| AG | 24 (54.5%) | 22 (37.9%) | 0.23 (0.04-1.21) 0.082 | 11 (30.6%) | 1.26 (0.32-4.98) 0.740 |
| AA | 18 (40.9%) | 26 (44.8%) | 0.29 (0.05-1.55) 0.147 | 21 (58.3%) | 2.02 (0.55-7.37) 0.287 |
| **Alleles** | N=88 | N=116 |  | N=72 |  |
| G | 28 (31.8%) | 42 (36.2%) | Reference | 19 (26.4%) | Reference |
| A | 60 (68.2%) | 74 (63.8%) | 0.76 (0.41-1.42) 0.395 | 53 (73.6%) | 1.58 (0.83-3.01) 0.167 |
| **Females** | N=60 | N=102 |  | N=42 |  |
| GG | 9 (15.0%) | 20 (19.6%) | Reference | 4 (9.5%) | Reference |
| AG | 26 (43.3%) | 49 (48.0%) | 0.84 (0.31-2.30) 0.736 | 21 (50.0%) | 1.99 (0.60-6.63) 0.261 |
| AA | 25 (41.7%) | 33 (32.4%) | 0.61 (0.22-1.72) 0.351 | 17 (40.5%) | 2.38 (0.69-8.19) 0.170 |
| **Alleles** | N=120 | N=204 |  | N=84 |  |
| G | 44 (36.7%) | 89 (43.6%) | Reference | 29 (34.5%) | Reference |
| A | 76 (63.3%) | 115 (56.4%) | 0.76 (0.45-1.27) 0.297 | 55 (65.5%) | 1.42 (0.83-2.42) 0.202 |

NDC= Non diabetic controls; T2DM= Type 2 Diabetes mellitus; DNR=Diabetic non-retinopathy; DR= Diabetic retinopathy; ^‡^Age adjusted OR and P value; significant values shown in **bold**; P_corr_= P value after bonferroni correction; ^†^P_corr_= 0.084 and **^††^**P_corr_= 0.052 respectively

Supplementary Table S3: Association of *SLMAP* rs17058639 C>T genotypes with clinical phenotypes (one-way ANOVA analysis)

| **Characteristics** | ***SLMAP* rs17058639 C>T** | | | **p-value** |
| --- | --- | --- | --- | --- |
|  | **CC**  **Mean±SD (N)** | **CT**  **Mean±SD (N)** | **TT**  **Mean±SD (N)** |  |
| **Mean Age (Yrs± SD)** | 54.0±11.15 | 53.08±10.95 | 53.76±10.40 | 0.770 |
| **Mean Glucose levels(mmole/lit)±SD** | 8.72±6.79 | 9.41±4.71 | 10.04±4.38 | 0.425 |
| **BMI (mean ±SD)** | 32.75±7.02 | 33.53±6.92 | 31.96±7.08 | 0.367 |
| **Creatinine level (micromole/lit) ±SD** | 78.36±43.72 | 68.49±27.41 | 71.03±27.52 | 0.121 |
| **Glycated hemoglobin (HbA1c) mean% ±SD** | 8.27±1.91 | 8.06±1.86 | 8.45±1.83 | 0.563 |
| **Total Cholesterol (mmol/lit) mean ±SD** | 4.83±0.85 | 4.94±1.11 | 4.65±1.05 | 0.338 |
| **HDL (mmol/lit) mean ±SD** | 1.26±0.32 | 1.28±0.37 | 1.15±0.31 | 0.165 |
| **LDL(mmol/lit)mean±SD** | 2.88±0.79 | 2.98±1.05 | 2.76±1.03 | 0.499 |
| **HDL/LDL ratio (mean ±SD)** | 0.47±0.19 | 0.50±0.29 | 0.47 ±0.20 | 0.678 |
| **Triglycerides (mmol/lit) mean±SD** | 1.55±0.79 | 1.67±1.24 | 1.73±0.98 | 0.599 |
| **Blood Urea Nitrogen (mmol/lit) mean±SD** | 6.03±3.56 | 5.20±2.47 | 5.90±3.94 | 0.152 |

Supplementary Table S4: Association of *SLMAP* rs17058639 C>T genotypes with progression of diabetic retinopathy

| ***SLMAP* rs17058639 C>T genotype** | **DNR (N=160)**  **N (%)** | **NPDR/ BDR (N=19)**  **N (%)** | **DNR vs NPDR/BDR**  **OR (95% CI) P** | **DME (N=49)**  **N (%)** | **OR (95% CI) P** | **PDR (N=10)**  **N (%)** | **OR (95% CI) P** |
| --- | --- | --- | --- | --- | --- | --- | --- |
| TT | 29 (18.1%) | 1 (5.3%) | Reference | 4 (8.2%) | Reference | 1 (10%) | Reference |
| CT | 71 (44.4%) | 8 (42.1%) | 3.28 (0.39-27.55) 0.274 | 20 (40.8%) | 2.13 (0.66-6.87) 0.207 | 2 (20%) | 0.77 (0.07-9.04) 0.837 |
| CC | 60 (37.5%) | 10 (39.1%) | 4.76 (0.58-39.11) 0.146 | 25 (51.0%) | 2.81 (0.88-8.94) 0.081 | 7 (70%) | 3.24 (0.37-28.50) 0.290 |
|  |  |  | P_trend_= 0.1058 |  | **P_trend_= 0.0425** |  | P_trend_=0.0849 |
| **Grouped analysis** |  |  |  |  |  |  |  |
| TT+CT | 100 (62.5%) | 9 (47.4%) | Reference | 24 (49.0%) | Reference | 3 (30%) | Reference |
| CC | 60 (37.5%) | 10 (39.1%) | 1.83 (0.70-4.82) 0.221 | 25 (51.0%) | 1.57 (0.81-3.04) 0.182 | 7 (70%) | 3.87 (0.93-16.08) 0.063 |

DNR=Diabetic non-retinopathy; DR= Diabetic retinopathy; NPDR=Non-proliferative diabetic retinopathy; PDR= Proliferative diabetic retinopathy; BDR= Background diabetic retinopathy; DME= Diabetic macular edema; P_trend_= P value for trend analysis.

Supplementary Table S5: Pair-wise linkage disequilibrium (Test of linkage disequilibrium for all pairs of loci)

| Pair of loci | **Non diabetic controls (NDC) (N=208)** | | |  | **T2DM patients without DR (DNR) (N=320** | | |  | **T2DM patients with DR (N=156)** | | |  |
| --- | --- | --- | --- | --- | --- | --- | --- | --- | --- | --- | --- | --- |
|  | Chi-square test value | P-value* | │D'│ Value | r^2^ | Chi-square test value | P-value* | │D'│ Value | r^2^ | Chi-square test value | P-value* | │D'│ Value | r^2^ |
| *SLMAP* _rs17058639_**SLMAP* _rs1043045_ | 195.05 | **0.0001** | 1.00 | 0.9584 | 303.77 | **0.002** | 1.00 | 0.9619 | 121.1850 | **0.0001** | 1.00 | 0.8045 |
| *SLMAP* _rs17058639_**SLMAP* _rs1057719_ | 199.25 | **0.0001** | 1.00 | 0.9787 | 307.7357 | **0.002** | 1.00 | 0.9744 | 120.1699 | **0.0001** | 0.9640 | 0.7696 |
| *SLMAP* _rs1043045_**SLMAP* _rs1057719_ | 199.31 | **0.0001** | 1.00 | 0.9791 | 311.8042 | **0.002** | 1.00 | 0.9872 | 146.8376 | **0.0001** | 1.00 | 0.9705 |

NDC= Non diabetic controls; T2DM= Type 2 Diabetes mellitus; DNR=Diabetic non-retinopathy; DR= Diabetic retinopathy; *Statistical estimates have taken after Yate’s continuity correction; significant values shown in **bold.**
